# Endogenous inclusion in the Demographic and Health Survey anthropometric sample: Implications for studying height within households^☆^

**DOI:** 10.1016/j.jdeveco.2021.102783

**Published:** 2022-03

**Authors:** Dean Spears, Diane Coffey, Jere R. Behrman

**Affiliations:** a University of Texas at Austin and the Research Institute for Compassionate Economics (r.i.c.e), United States of America; b University of Pennsylvania, United States of America

**Keywords:** Demographic and Health Surveys, Birth order, Fertility, Height, India, Selection into identification

## Abstract

Development economists study both anthropometry and intra-household allocation. In these literatures, the Demographic and Household Surveys (DHS) are essential. The DHS censors its anthropometric sample by age: only children under five are measured. We document several econometric consequences, especially for estimating birth-order effects. Child birth order and mothers’ fertility are highly correlated in the age-censored anthropometric subsample. Moreover, family structures and age patterns that permit within-family comparisons of siblings’ anthropometry are unrepresentative. So strategies that could separate birth order and fertility in other data cannot here. We show that stratification by mother’s fertility is important. We illustrate this by comparing India and sub-Saharan Africa (SSA). Children in India born to higher-fertility mothers are shorter, on average, than children of lower-fertility mothers. Yet, later-born children in India are taller, adjusted for age, than earlier-born children of the same sibsize. In SSA, neither of these associations is large.

## Highlights

• The DHS censors its anthropometric sample by age: only children <5yrs are measured.
• We document econometric consequences, especially for studying birth-order effects.
• Strategies that could separate birth order and fertility in other data cannot here.
• We show that stratification by mother’s fertility is important.
• We illustrate this by comparing India and sub-Saharan Africa.

## 1 Introduction

For developing economies, anthropometric measures, particularly height, are important markers of human capital (Behrman and Deolalikar, 1988, Victora et al., 2008, Deaton, 2013, Hoddinott et al., 2013, Black et al., 2017). For example, stunting – short height compared to well-nourished populations – is the primary indicator used in *The Lancet* estimates that  250 million children under five years of age are at risk of not fulfilling their potential (Black et al., 2017). Households are important sites of allocation and production (Strauss and Thomas, 1995). So an enduring focus of the development economics literature studies the effects of household structures and processes on anthropometric outcomes (Behrman, 1988). For this topic, Demographic and Health Surveys (DHS) are uniquely important. For many countries, the DHS is the only source of population-representative anthropometric data.

The DHS censors its height (and other anthropometric measures) sample by age. Although the DHS asks about *early-life mortality*, for example, for all children yet born to a mother, it measures the *height* only of children who were born in the five years before the day of the interview. One immediate consequence is that only younger children of mothers are in the height subsample. Another consequence is that the subset of families with multiple children whose heights can be compared differs from the full population. For example, they have different age distributions and shorter birth spacing, an important (but methodologically challenging) predictor of child outcomes (Potter, 1988). A third consequence is that the correlation between children’s birth orders and their mothers’ fertility is stronger in the height subsample than in the full population. This matters, in part, because the magnitudes and even the signs of correlations between fertility and socioeconomic status range widely across developing-country populations (Vogl, 2015).

Thus, the fact that the DHS censors the height sample by age matters for researchers using within-family empirical strategies, and especially for those studying birth order. In particular, (Blake, 1989)’s recommendation that studies of birth order should be stratified by number of siblings is critical when using the DHS’ age-censored anthropometric subsample. Despite the importance of both birth order and nutritional outcomes, to our knowledge these implications for using the DHS have not previously been comprehensively documented.

In Section 2, we discuss the DHS data that we use. We show that the age-censoring of the anthropometric subsample introduces econometrically-relevant properties that differ from the full population. Throughout, we compare India and sub-Saharan Africa (SSA) because these two populations are at different points in the demographic transition: in India, high fertility is uncommon and negatively-selective for health and wealth, but in SSA, high fertility is common and less negatively-selective (indeed, it is positively-selective by some measures). In Section 3, we document this important background for any effort to estimate an effect of birth order in this context. Because a mother having higher fertility, rather than lower fertility, implies more socioeconomic disadvantage within India than within SSA, family size must be accounted for.

Section 4 presents the methodological challenge that is our focus: we show that, even in regressions where there should be no “effect” of birth order, the endogenous censoring of the DHS height subsample exacerbates the omitted variable bias threat from fertility. Age censoring also undermines some common empirical strategies that could resolve this issue with full birth histories but cannot in the height subsample. Our results point towards stratification by mothers’ fertility as an important procedure.

We use stratification in non-parametric summaries in Section 5. We show that Indian children born to higher-fertility mothers are notably shorter, on average, than Indian children of lower-fertility mothers. And yet, later-born children in India of a given sibsize are taller, adjusted for age, than earlier-born children of the same sibsize. This pattern is in striking contrast with evidence of later-born disadvantage in other studies of birth order, especially in developed countries (*e.g.* Black et al., 2005). In SSA, neither of these associations is very large. Finally, in Section 6 we present a practical application: a reinterpretation of a study published in the *American Economic Review* of height and birth order in the DHS.

## 2 Data

We compare India and SSA, two populous regions at different points in the fertility transition. For India, we use the 2005–6 Indian DHS, which is called the National Family Health Survey-3 by the Government of India. For SSA, we use 27 comparably-timed DHS survey rounds from 25 countries. To study birth order, we exclude multiple births such as twins.

We use three samples from the Indian and SSA DHS birth histories:

• **Full birth history:**n=987,447. These are all children ever born alive (prior to the survey) to the mothers surveyed by the DHS. 83% of these do not have measured height.
• **Main height sample:**n=166,153. These are children under 60 months old who are alive at the time of the survey and who have their heights measured.
• **Mother-fixed-effects subsample:**n=80,785. These are children under 60 months old who are alive at the time of the survey, who have their heights measured, and who have at least one sibling who also meets these criteria.

Table 1 describes the make-up of the main height sample by birth order and sibsize. Panels (a) and (b) show that the age-censoring makes sibsize and birth order highly correlated in the main height sample. The correlation between birth order and sibsize is 0.98 in the age-censored main height sample, compared with 0.63 in the full birth history of children ever born in the same DHS rounds. In the main height sample, 72% of measured children are the last born to their mother and 97% are the last or next-to-last born at the time of the survey, compared with 28% and 50% in the full birth history.

**Table 1:** Structure of main height sample: Counts of observations by demographic category.

Panel (a): Counts by sibsize and birth order in India					
	Sibsize at time of survey				
	1	2	3	≥4	all sibsizes
birth order:					
1	8,680	4,210	457	23	13,370
2		9,261	2,352	309	11,922
3			5,346	1,523	6,869
≥4				9,811	9,811
all birth orders	8,680	13,471	8,155	11,666	41,972

Panel (b): Counts by sibsize and birth order in SSA					
	Sibsize at time of survey				
	1	2	3	≥4	all sibsizes
birth order:					
1	17,413	7,287	901	21	25,622
2		16,388	6,434	700	23,522
3			13,866	5,827	19,693
≥4				55,344	55,344
all birth orders	17,413	23,675	21,201	61,892	124,181

Panel (c): Counts of available within-family comparisons									
		Child’s birth-order-distance from last born							
heights per sibship		0	1	2	3	4	5		total
1		80,737	4,419	196	15	1	0		85,368
2		35,507	34,932	1,420	71	2	2		71,934
3		2,887	2,870	2,877	45	3	0		8,682
4		41	40	40	41	2	0		164
5		1	1	1	1	1	0		5
total		119,173	42,262	4,534	173	9	2		166,153

*Note:* “Child’s birth-order-distance from last born” is (sibsize at the time of the height measurement — birth order). So, because $\frac{119,173}{166,153}=72\text{\%}$ of the main height sample has a value of 0, 72% of the main height sample is the last born to their mother (these are the diagonal elements in Panels (a) and (b)). “Heights per sibship” is the number of measured heights in a child’s sibship; only children with a value of 2 or greater could be in the mother-fixed-effects subsample. $\frac{85,368}{166,153}=51\text{\%}$ of the main height sample is ineligible for the mother-fixed-effects subsample for this reason.

Another implication of Table 1 is that, within any sibsize, the heights of *last-born and next-to-last-born children* can be compared. But comparing first-borns with third-borns in sibships of 3, for example, is constrained by the age censoring. Panel (c) focuses on children’s eligibility for the mother-fixed-effects subsample. Consider using these data to answer the seemingly simple question of whether third-born children tend to be shorter than their first-born *siblings*. In the framework of Panel (c), a first-born child is 2 or more from last-born, if in a sibsize of 3 or more. Table 1 shows that such a child is an unusual observation in these age-censored data. In India, only about 3% of the measured first-borns are in sibsizes of 3 or more; less than 6% of measured children in a sibsize of 3 are first-born. Less than 5% of third-borns in the height data have a measured first-born sibling (even though, by definition, 100% of them had one born alive).

The few large families in which early-borns are young enough to be measured are negatively selected for their families’ short birth spacing. For example, controlling for child age and sex, third-borns in India in sibships of 3 with a measured first-born sibling are shorter than third-borns without a measured first-born sibling by a gap almost as large as that between urban and rural Indian children.^1^ Short birth spacing has important consequences, itself, and is correlated with other disadvantages.

Additionally, these measured pairs are at the two extreme ends of the five years of measured age. The average first-born and third-born in the height subsample are both 28 months old (the mortality-weighted middle of the 0–59 range) but are 51 and 8 months old, respectively, among the selected subset that can be compared with one another within households. Age predicts height-for-age. These facts illustrate that the age-censored height sample is not constructed to permit such within-sibship comparisons.

## 3 Background

Birth order is necessarily correlated with other variables that are correlated with child outcomes, including child age, mothers’ fertility, mothers’ age at birth, and birth spacing. These identification challenges have been well-studied. As [9] writes to introduce an “outline of confounding factors in the analysis of birth order:” … “most findings appear to involve highly selective reporting of what are, in reality, sibsize, period, parental background, child-spacing, and other selection effects for which controls have not been instituted” (p. 300). Among these, sibsize has received special attention in the economics literature because higher-fertility mothers are different, on average, than lower-fertility mothers (Black et al., 2005). In an early study of birth-order effects in the economics literature, [6] note this problem, citing [17] and observing that “many studies … fail to control for family size and family background. Later birth orders, for example, are only observed for larger families that have different child quantity/child quality trade-offs. If so, then birth-order effects from interfamilial data may be reflecting only differential child quality/quantity shadow prices across families and not within-family birth-order effects” (p. S131).

Average fertility is lower in India (Das Gupta and Mari Bhat, 1997, Drèze and Murthi, 2001) than in SSA (Caldwell and Caldwell, 1987, Kohler and Behrman, 2014). For the 2005–2010 interval – which is the period that includes the Indian DHS that we use here – the UN World Population Prospects estimates that SSA’s total fertility rate was 5.4 live births per woman, compared with 2.8 in India.^2^ The relationship between fertility and socioeconomic status is understood to be positive in some present and past populations and negative in others (Schultz, 1981). [28] uses the same DHS data source that we study to document that, over recent decades, the gradient between fertility and economic status in developing countries has slowly switched from positive to negative. India and SSA are at different points in this transition.

**Fig. 1:**
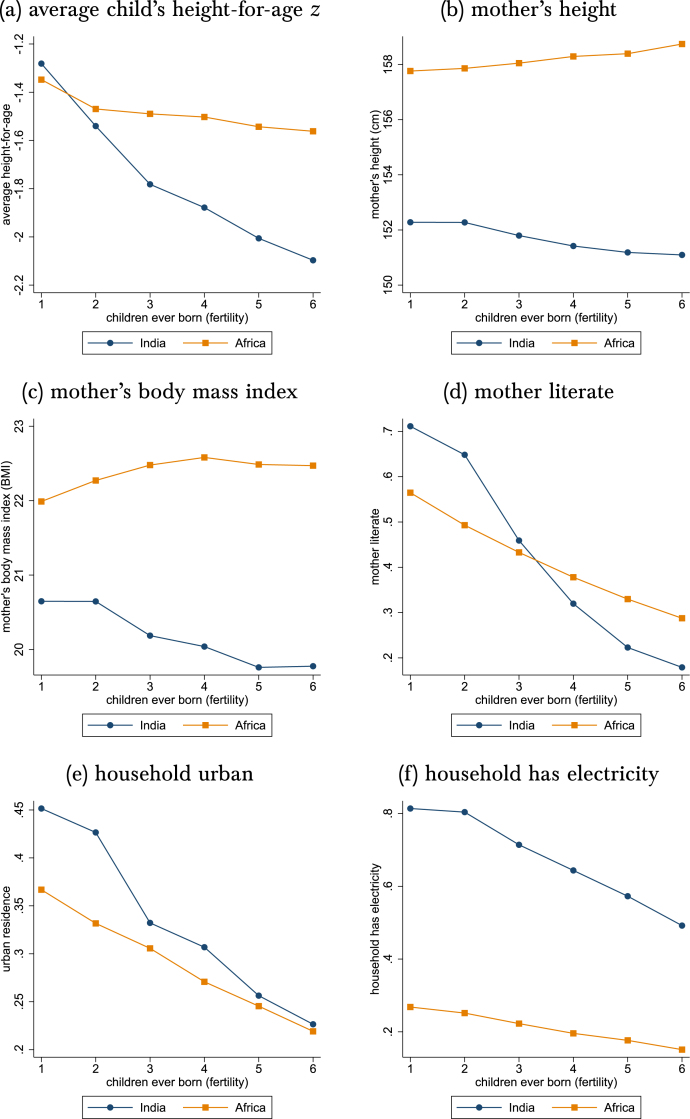
Background: Higher-fertility mothers are more disadvantaged in India, relative to lower-fertility mothers in India, than higher fertility mothers are in SSA, relative to lower-fertility mothers in SSA. *Note:* Observe that in every panel, the India line slopes more negatively than does the SSA line. In all panels, the sample is the same in Fig. 2, described in Section 2 as our “main height sample.”.

Fig. 1 documents that an omitted-variable-bias threat from sibsize is present among the children whose heights we study here. Fig. 1 shows that higher fertility predicts greater disadvantage by more in India than in SSA, in the sense of difference-in-differences.^3^ Panel (a) is particularly noteworthy, because it plots the average height-for-age z-score of all of a mother’s measured children. Height is steeply decreasing in sibsize in India. In SSA, mothers who have more children are taller and have more body mass, on average; in India they are shorter and have less body mass. So an omitted-variable threat from endogenous fertility is an observed property of these populations.

## 4 Example: “Effects” of birth order where there are none

Suppose for example that larger families were poorer and had children in worse health, but within each family all children were exactly equally healthy. A regression including children from multiple families of health on only birth order would find a negative association between being later-born and health (i.e., the regression would suggest an advantage for earlier-birth-order children). But this finding would be a spurious artifact of the bias created by the key omitted variable of sibsize.

Holding sibsize constant may be both especially important and especially challenging when a sample is age-censored to young children, as is the DHS anthropometric subsample. Techniques in the literature that are thought to account for a group-level omitted variable can fail when selection into the group (in this case, into a group of siblings young enough to have measured height) is endogenous (compare Ardington et al., 2009).

Table 2 uses the India and SSA DHS samples described in Section 2 to run regressions where birth-order indicators are the independent variables. The dependent variables are mother’s height (in Panels A and B) and mother’s literacy (in Panels C and D), both measured by the DHS at the fixed time of the survey. For these unusual regressions, we know *a priori* that there is zero causal effect of birth order on these dependent variables, because the “outcomes” must be the same for all siblings. As standalone research, these regressions make no sense. But they illustrate the challenges of using the height subsample. If a regression with such a specification does not yield a zero coefficient, we should investigate whether there exists a threat to identification.

**Table 2:** Example regressions, which should have zero coefficients, of fixed properties of mothers on birth order.

**inclusion in sample:**	(1)	(2)	(3)	(4)	(5)		(6)	(7)	(8)	(9)	(10)
height subsample?	no (full)	yes	no (full)	yes	yes		no (full)	yes	no (full)	yes	yes
birth order	≤3	≤3	≤3	≤3	≤2		≤3	≤3	≤3	≤3	≤2
sibsize	any	any	any	any	2 only		any	any	any	any	2 only
measured children per mother	any	any	any	any	2 only		any	any	any	any	2 only
											
	Panel A: India, dependent variable is mother’s height (cm)						Panel B: SSA, dependent variable is mother’s height (cm)				
birth order 2	−0.0485***	−0.0737	−0.0009	0.0714	0.0000		0.0735***	0.174**	−0.0070**	0.156*	0.0000
	(0.0105)	(0.0609)	(0.0013)	(0.0701)	(0.0000)		(0.0103)	(0.0565)	(0.0021)	(0.0709)	(0.0000)
birth order 3	−0.342***	−0.519***	0.0001	0.135			0.147***	0.353***	−0.0076${}^{†}$	0.257*	
	(0.0222)	(0.0856)	(0.0036)	(0.120)			(0.0169)	(0.0703)	(0.0045)	(0.101)	
sibsize indicators	no	no	yes	yes	stratified		no	no	yes	yes	stratified
n (live births)	188,948	31,978	188,948	31,978	7,518		347,918	68,360	347,918	68,360	12,758
corr.: birth ord. & sibsize	0.27	0.80	0.27	0.80	0.00		0.23	0.83	0.23	0.83	0.00
											
	Panel C: India, dependent variable is mother’s literacy						Panel D: SSA, dependent variable is mother’s literacy				
birth order 2	−0.0437***	−0.0748***	−0.0003*	0.0140*	0.0000		−0.0346***	−0.0598***	−0.0005***	0.0315***	0.0000
	(0.000973)	(0.00495)	(0.0001)	(0.00604)	(0.0000)		(0.000668)	(0.00389)	(0.0001)	(0.00485)	(0.0000)
birth order 3	−0.157***	−0.250***	0.0001	0.0284**			−0.0699***	−0.115***	−0.0003	0.0688***	
	(0.00234)	(0.00739)	(0.0003)	(0.0104)			(0.00112)	(0.00477)	(0.0003)	(0.00718)	
sibsize indicators	no	no	yes	yes	stratified		no	no	yes	yes	stratified
n (live births)	195,260	31,982	195,260	31,982	7,508		451,379	68,353	451,379	68,353	12,766
corr.: birth ord. & sibsize	0.27	0.80	0.27	0.80	0.00		0.23	0.83	0.23	0.83	0.00

*Note:* For clarity of interpretation, the data are restricted to children of birth orders 1, 2, or 3 only. Otherwise, columns 1, 3, 6, and 8 use the full birth history. Columns 2, 4, 5, 7, 9, and 10 use the main height sample, with columns 5 and 10 further restricted to pairs of measured siblings in sibsizes of 2. Standard errors are clustered by survey primary sampling unit (PSU). “corr.: birth ord. & sibsize” is the correlation between birth order and sibsize in that panel and column’s subsample. Two-sided p-values: †p<0.1; * p<0.05; ** p<0.01; *** p<0.001.

In Table 2, columns 1 and 3 (and the corresponding columns in Panels B and D) use the full birth history. Columns 2, 4, and 5 use only the height subsample; column 5 further restricts the sample to sibling pairs in sibsizes of two with both siblings measured, to implement stratification by sibsize. Only stratification by sibsize yields a zero coefficient on birth order — although including sibsize covariates comes close in the full birth history.

In the first two columns of each panel, sibsize is not controlled at all. Compare these results with the fertility gradients in Fig. 1. Increasing birth order has *negative* coefficients for mother’s height in India but *positive* coefficients in SSA, matching the slopes in panel (b) of Fig. 1. Moving from the first to the second column of each sample preserves the lack of sibsize covariates while restricting the sample from the full birth history to the main height sample. In each case, the coefficients increase in absolute magnitude, moving away from zero as the sample moves from the full birth history to the height subsample. This is because the correlation between birth order and sibsize is larger in the height subsample than in the full birth history. This makes accounting for endogenous sibsize even more important.

In the next two columns of each panel, indicators for sibsize are included as regression covariates. Columns 3 and 8 use the full birth history; columns 4 and 9 use the height subsample. If the full birth history is used, the estimate comes close to the correct zero coefficient. But in the height subsample, the coefficients are positive and large, and smaller in India than in SSA (more negative in India in a difference-in-differences sense). This is because of selection into the height subsample. If a mother has any living children young enough to be in the height subsample, then the last-born child (for whom birth order necessarily equals sibsize) is always included. Conditional on sibsize, earlier-born children are included only in cases of short birth spacing. Shorter birth spacing predicts worse outcomes for children. So *once sibsize is conditioned upon*, the presence of earlier-birth-order observations is a marker of disadvantage. This yields the spurious positive coefficients on later birth order in columns 4 and 9. Columns 3 and 8 do not show these positive coefficients because their sample is not age-censored.

Finally, columns 5 and 10 further restrict the height subsample to stratify down to sibsize of 2. Here, the regression coefficients are correctly zero. Table 2 shows that merely including sibsize indicators is not sufficient in the height subsample.

Might mother fixed effects – which would compare (some) children with their own siblings – work instead? In the regressions in Table 2, mother fixed effects cannot be estimated: the dependent variables are constant properties of mothers. For other situations, Section 2 has already demonstrated that mother fixed effects cannot plausibly be used to study birth order in the DHS height subsample. The subset of measured children with measured siblings – especially two or more birth-order places apart – is both small and unrepresentatively selected.

**Fig. 2:**
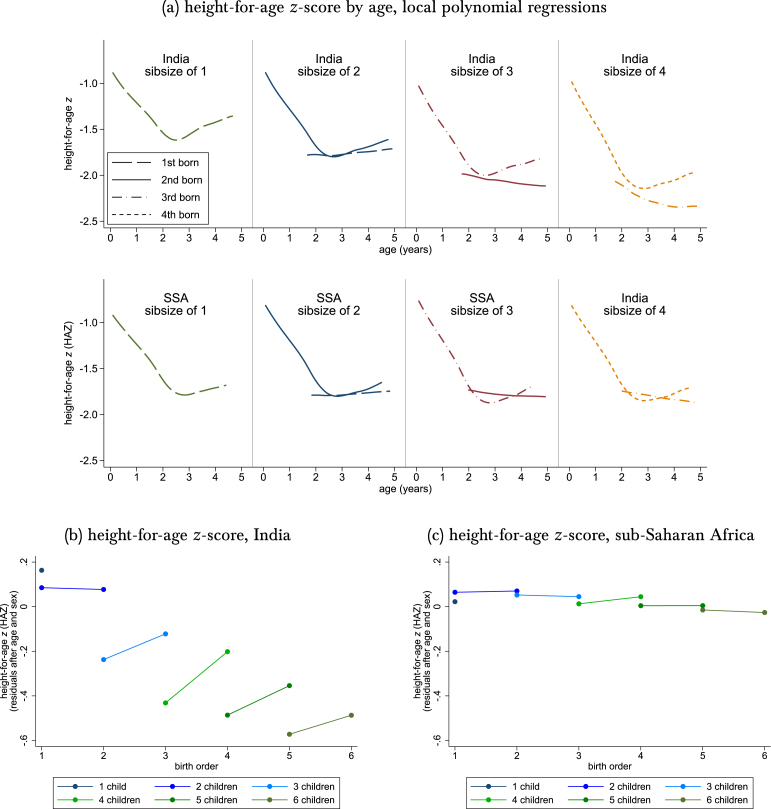
Height-for-age z: Non-parametric stratification by sibsize and birth order. *Note:* Data are the main height sample, but plotting only the 97% of height observations that are last-born or next-to-last-born (see Table 1). The count of children by which the sample is split is the number of children ever born to the mother by the time of the interview, which is the variable on the horizontal axis of each panel of Fig. 1. Panel (a) uses an Epanechnikov kernel and a 9-month bandwidth and restricts ages (in months) to within the 2.5th to 97.5th percentile within each combination of India/SSA, birth order, and sibsize (panels (b) and (c) impose no age restrictions). Residuals used in panels (b) and (c) are from one regression of HAZ on 120 age-by-sex indicators and no other covariates, computed in the entire main height sample, without restrictions or stratification..

## 5 Stratification results

How can researchers investigate the relationship between birth order and height, given these challenges? The simplest place to start is to plot stratified, non-parametric summary statistics. Fig. 2 does so. Here, and in the rest of this paper, children’s height is operationalized as a height-for-age z-score (HAZ) within sex and age-in-months, based on international WHO norms (World Health Organization et al., 2006). For clarity, only the last or next-to-last births to a mother are included in Fig. 2: 97% of the main height sample is either last-born or next-to-last born. Section 2 documented that the remaining 3% is unrepresentative.

Panel (a) plots local polynomial regressions of HAZ on age. No further controls, residualization, or restrictions are used beyond stratifying the sample by birth order and sibsize. There is a well-studied relationship between HAZ and age in children under two in developing countries (Victora et al., 2010, Aiyar and Cummins, 2021): mean HAZ falls over the first two years of life and flattens (as a function of age) at age two. This pattern is visible in Panel (a). Panel (a) also shows that not all ages can be observed for all combinations of birth order and sibsize: in sibsizes of 2, for example, no first-borns were measured when they were 6 months old.

In SSA, neither birth order nor sibsize is predictive of HAZ: within a sibsize, at the same age, HAZ lines for different birth orders overlap. India is different. Across sibsizes, there is a downward trend: children from larger sibsizes are shorter, on average. Within a sibsize, at the same age (and where the same age is measured for both birth orders), later-born children are taller, on average. More specifically, in sibsizes of 2 there is no evidence that first-borns are taller than second-borns. Fig. 2 shows that in sibsizes of 3 and 4, later-borns are taller at the same age.

The rest of this paper considers how to compress the information in Panel (a) of Fig. 2 into informative means, regression coefficients, and other statistics. One clear message from Fig. 2 is that any appropriate empirical strategy must account for the declining pattern of HAZ in sibsize that is apparent for India but not for SSA.

Another important lesson of Panel (a) in Fig. 2 is that, conditional on sibsize, age is predictive of birth order. Because HAZ, too, is predicted by age, age is a potential confounding factor. Age is endogenously selected in any within-household comparison, as detailed in Section 2. So any credible empirical strategy must robustly account for child age.

In the birth-order literature, there is a standard non-parametric tool described in detail by [9] and used more recently by [7]: a plot of mean outcomes by birth order and sibsize. This is the next step towards reducing the information in Panel (a) into regression coefficients. Panels (b) and (c) present such a plot. The horizontal axis is sibsize. Unlike investigations of birth order in the literature that study outcomes at a fixed age, here we must first account for age. The vertical axis is the average residual of children’s height-for-age z-scores after regression on a set of 120 age-in-months × sex categories (this is the resolution of the z-score reference tables).

As in Panel (a), three patterns are clear in Panels (b) and (c): (1) that in India higher sibsize is associated with shorter child height; (2) that there is no later-born height disadvantage in India; and (3) that neither of these patterns is pronounced in SSA. In other words, in India, children *with more siblings* are shorter than children *with fewer siblings* at the same age.

## 6 Application: Comparing birth order gradients

What would we learn if we attempted to summarize Fig. 2 with regressions? [19] – hereafter JP – have investigated birth-order gradients in height in India and SSA. Their main result reports that HAZ is more negatively associated with birth order in India than in SSA. JP interpret this correlation as evidence that parents in India discriminate against later-born children within households.

We apply the methodological observations of this paper to ask whether the correlations between birth order and sibsize in India and SSA can be understood as causal effects. More specifically, we ask what JP would have found if they had stratified by sibsize.

Our purpose here is to illustrate the importance of endogenous fertility in the age-censored DHS child height subsample — not to assess each econometric choice of JP. JP recognize the potential that sibsize could be an important confounder. They discuss the threat and implement several robustness checks. Here we show how these strategies are limited by the structure of the available data.

### 6.1 Regression empirical strategy

JP estimate the following regression specification, their Eq. (1):

(1) $$\begin{array}{ccc} HAZ_{imc} & = & \alpha_{1}{\mathrm{India}}_{c}+\alpha_{2}{\mathrm{India}}_{c}\times {\mathrm{second - born}}_{imc}\\ & & +\;\alpha_{3}{\mathrm{India}}_{c}\times {\mathrm{third - or - later - born}}_{imc}\\ & & +\;\beta_{1}+\beta_{2}{\mathrm{second - born}}_{imc}\\ & & +\;\beta_{3}{\mathrm{third - or - later - born}}_{imc}+\gamma X_{imc}+{ɛ}_{imc}, \end{array}$$

where i indexes children, m indexes mothers, c indexes the country, and ${\mathrm{India}}_{c}$ is an indicator that is 1 for Indian observations and 0 for SSA. The coefficients of interest are $\alpha_{2}$ and $\alpha_{3}$, which are the difference-in-difference estimators for the birth-order gradients. $\beta_{2}$ and $\beta_{3}$ are average HAZ differences between first-borns (the omitted group) and second- and third-borns respectively, in SSA. Sibsize is not accounted for in regression Eq. (1), nor in JP’s corresponding Figure 2. In this way, Eq. (1) is like the uncontrolled specifications in columns 2 and 7 of Table 2.

JP are aware that sibsize could be an omitted variable in their analysis. They write: “Higher birth order children are more likely to come from larger families, and family size could be correlated with child height; family size could affect child height via its effect on the available resources per child, plus larger families tend to be poorer” (p. 2609; they do not, however, raise the possibility that this correlation may be *different* between India and SSA, where larger families are *advantaged* by some relevant measures). They argue against including a regression control for sibsize, as observed at the time of the survey, because women’s childbearing careers will often be incomplete: “the nature of DHS sampling implies that a large fraction of households in our sample have not completed childbearing… our regressions cannot control for total family size in general, raising an omitted variable bias concern”. This is a different concern about including sibsize as a covariate than we raise in Section 4: our concern there was that inclusion in the height sample is negatively selective for birth orders that are early, relative to their sibsize. Here, JP note another constraint for any effort to study birth order and height in the DHS: By the construction of a subsample that only includes young children, completed sibsize for many children is unobserved (and, in fact, undetermined) at the time of the survey. JP’s proposed solution, which we adopt below, is to make a further restriction of the sample to mothers who they interpret to be likely to have completed fertility.

In many other birth-order studies, age is held constant in the dependent variable, so the time period of interest differs across siblings: for example, [7] study educational attainment by age 25, [13] study mortality in the first month of life, and [10] study inputs at specific gestational and early-life ages. When studying height with the DHS, however, age cannot be held constant. The DHS measures height at one point in time per family, so children are measured at different ages. Because later-born children are younger, and therefore more likely to be on the declining HAZ path shown in Fig. 2, they appear taller if age is not controlled. Our $X_{imc}$ therefore includes 119 age-in-months-by-sex indicators, which is the resolution of the WHO reference tables.

Eq. (1) considers differences in height by birth order relative to first-born children, the omitted category. And yet, Section 2 suggests that first-borns can only be compared plausibly, in these data, to second-borns. Section 2 shows that almost 40% of the children in the main height sample are third-born or later. In SSA, where the average women had almost twice as many children as in India during this time, 50% of the sample is of sibsize 4 or more and 45% are fourth-born or later. Because JP topcode birth order at “3 or greater”, such larger sibsizes and later-born children contribute little to JP’s identifying variation.

**Table 3:** Application: Regressions of height-for-age z-score on birth order.

	(1)	(2)	(3)	(4)	(5)	(6)	(7)	(8)
**inclusion in sample:**								

measured children per mother	any	any	2	2	2	2	2	≥2
sibsize	any	any	any	2	2	3	3	any
birth orders	any	any	any	1 and 2	1 and 2	2 and 3	2 and 3	any

Panel A: No further sample restrictions								

birth order 2 ×India	−0.151***	−0.0066	−0.148***	−0.0577	0.00962			−0.0437
	(0.0246)	(0.0311)	(0.0349)	(0.0405)	(0.0404)			(0.0365)
birth order 3+ ×India	−0.386***	0.105*	−0.382***			0.0689	0.129*	0.0176
	(0.0251)	(0.049)	(0.0393)			(0.0564)	(0.0557)	(0.0577)
India	0.0871***		0.00536	0.0110		−0.282***		
	(0.0212)		(0.0326)	(0.0332)		(0.0431)		
birth order 2	0.0326*	−0.0153	−0.102***	−0.241***	−0.919***			−0.219***
	(0.0154)	(0.0214)	(0.0231)	(0.0527)	(0.0736)			(0.027)
birth order 3+	−0.0444***	−0.0912**	−0.160***			−0.476***	−1.021***	−0.445***
	(0.0134)	(0.0319)	(0.0233)			(0.0742)	(0.0969)	(0.042)
sibsize ×India	no	yes	no	no	no	no	no	no
mother fixed effects	no	no	no	no	yes	no	yes	yes
n (measured children under 5)	166,153	166,153	71,934	20,374	20,374	14,112	14,112	80,785

Panel B: Sample restricted to “completed fertility” subsample								

birth order 2 ×India	−0.163***	−0.012	−0.152*	−0.0544	0.0215			−0.0492
	(0.0428)	(0.053)	(0.0590)	(0.0723)	(0.0721)			(0.0640)
birth order 3+ ×India	−0.444***	0.052	−0.421***			0.0701	0.128	−0.0002
	(0.0402)	(0.076)	(0.0617)			(0.0857)	(0.0834)	(0.0931)
India	0.116**		0.0405	0.0444		−0.208***		
	(0.0364)		(0.0547)	(0.0559)		(0.0621)		
birth order 2	0.0454	−0.0064	−0.129*	−0.249**	−0.834***			−0.223***
	(0.0349)	(0.0459)	(0.0503)	(0.0900)	(0.117)			(0.058)
birth order 3+	−0.0444	−0.0436	−0.192***			−0.424***	−1.000***	−0.412***
	(0.0296)	(0.0638)	(0.0494)			(0.120)	(0.152)	(0.084)
sibsize ×India	no	yes	no	no	no	no	no	no
mother fixed effects	no	no	no	no	yes	no	yes	yes
n (measured children under 5)	67,194	67,194	29,790	7,316	7,316	5,016	5,016	33,822

*Note:* In all columns the dependent variable is the child’s height-for-age z-score. Column 1 of Panel A uses the main height sample; other columns are restricted to subsets of this sample, as noted in the table. All columns include fixed effects for 119 age-in-months by sex categories. Standard errors are clustered by survey primary sampling unit (PSU). Two-sided p-values: * p<0.05; ** p<0.01; *** p<0.001.

### 6.2 Regression results

Throughout, we have used the same set of Indian and SSA survey rounds as JP.^4^ Table 3 investigates the consequences of stratifying by sibsize for these regression results. Panel A uses the full main height sample. We build upon JP’s isolation of a subsample of families that they identify as likely to have completed childbearing. Panel B uses the “completed fertility” subsample as identified and named by JP.^5^ For these families, JP interpret sibsize at the time of the survey to be an adequate measure of final sibsize.

Column 1 of Panel A is our replication of JP’s main result, presented in their column 2 of their Table 2. Our estimates in Column 1 are quantitatively similar to what JP find: First-born children in India with height measured in the DHS are taller, on average, than first-born children of the same age in SSA; HAZ differences by birth order in SSA are small; and the average later-born child with height measured in the DHS in India is shorter than the average first-born with height measured. In Section 4 and elsewhere, we have discussed the inadequacy of merely adding covariate controls for sibsize × India. But such regression controls do offer a simple response to any concern that fertility is an omitted variable. So, for completeness, we add these covariates in column 2. Consistent with the non-parametric results of Fig. 2, the apparent negative interaction from column 1 is eliminated or reversed in column 2.

The next step is to stratify by sibsize. To permit a clear comparison with our stratified results, column 3 restricts the sample to the minority subsample with two measured height observations per family. In the four combinations of columns 1 and 3 of Panels A and B, the interactions of interest between birth order and an India indicator are quantitatively stable. The non-interacted coefficient on India becomes much smaller because this coefficient reflects first-borns, many of whom are now excluded. Restricting the sample to sibling pairs excludes all first-borns in sibsizes of 1: these first-borns with no siblings can teach us little about within-household discrimination by birth order, but they pose a threat to identification because, as Fig. 2 shows, in India they are 0.5 standard deviations taller than first-borns with siblings.

Columns 4 through 7 account for sibsize by holding it constant in stratified samples: columns 4 and 5 restrict the sample to children in sibsizes of 2; columns 6 and 7 restrict the sample to children in sibsizes of 3. 91% of Indian families in the sample in which a first-born and a second-born both have measured height are from a sibsize of 2. Columns 5 and 7 further include mother fixed effects. Stratifying by sibsize eliminates or reverses the negative interaction between India and later birth order, with or without mother fixed effects.^6^

Finally, for completeness and despite concerns raised in Section 4, column 8 adds mother fixed effects to the specification in column 1, preserving other aspects of the sample and specification. There is no evidence of a negative interaction.

In short, sibsize makes an important difference. Table 3 essentially replicates JP’s finding in column 1 without accounting for endogenous fertility. But there is no evidence in the DHS that India shows a special later-born disadvantage once sibsize is held constant — whether by direct regression control (column 2), by stratification (columns 4 through 7), or by mother fixed effects (columns 5, 7, and 8). This is true whether all available observations are used (Panel A) or whether the sample is restricted to the completed-fertility subsample (Panel B). Consistent with the non-parametric evidence of Fig. 2, the interaction for third-borns is robustly positive.^7^

### 6.3 Why are these results different?

Because of the correlation between child height and sibsize, the negative correlation between birth order and child height in India cannot be interpreted as a negative effect of birth order. Some results even suggest a positive effect. Our conclusion, however, is that the structure of the available DHS data prevents researchers from being able to interpret these results with confidence as an effect of birth order on child height.

Beyond its econometric importance for internal validity, the age-censoring of the DHS height subsample also influences the external validity of these results. JP propose that their results are due to a *within-family* process of discrimination. They write in their abstract: “We posit that India’s steep birth order gradient is due to favoritism towards eldest sons, which affects parents’ fertility decisions and resource allocation across children”. But, as Table 1 shows, the DHS is not structured to study within-family processes. For 71% of the families in the main height sample (corresponding to over half of the child-level observations), only one child’s height is observed. Only 2% of families have measured height from three or more children.

One question is why JP’s analysis – despite its extended set of robustness checks – suggests a negative relationship between height and birth order, particularly in a robustness check with mother fixed effects. It is understandable that an econometrician might consider mother fixed effects as a solution for confounding heterogeneity at the sibship level. The fact that sibsize can be an omitted variable in a study of birth order is simple and straightforward. The failure of mother fixed effects, in an age-censored subsample, for two estimates of the effect of birth order in two populations, on an outcome measured at different ages for different siblings, is not. We propose that the age-censoring of DHS data causes an instance of what [21] have recently defined and described as a “selection into identification” problem. Selection into identification occurs in cases of parameter heterogeneity, when a fixed-effects subsample is misleadingly different from the population of interest.

In this case, using mother fixed effects selects for very short birth spacing (Behrman, 1988). Short birth spacing predicts low birthweight among babies; low birthweight predicts disadvantage. To clarify, short birth spacing is not an *omitted variable* here to be controlled, but rather is an *interactor* with birth order that marks heterogeneity in the parameter of interest. Because birth spacing is a *difference* between two siblings and is a correlate of age, it violates the *strict exogeneity* requirement of fixed effects (Wooldridge, 2010).^8^

A separate example of the consequences of mother fixed effects concerns SSA. JP’s summary statistics and main Eq. (1) find that second-borns in SSA are slightly taller than first-borns in SSA. But JP’s fixed-effects column claims that second-borns in SSA are much shorter than first-borns in SSA (by even more than the overall India-SSA height gap). Fixed effects claim an even larger disadvantage for SSA third-borns. These fixed-effects results are incompatible with JP’s main results.

Of course, mother fixed effects can be a useful tool in data without the constraints of the DHS anthropometric subsample. [13] find a relative later-born *advantage* in India when studying consequences for early-life mortality in the full birth histories of the same set of DHS surveys that we and JP use. They find similar results whether they stratify by sibsize, use mother fixed effects, or include regression controls for sibsize directly. DHS mortality data are not censored by child age. Nor are age controls involved, because infant and neonatal mortality rates are age-specific. Finally, DHS birth histories include children of mothers whose fertility is long-completed. Coffey and Spears present evidence that such a later-born neonatal survival advantage in India reflects improvements in maternal nutrition over the course of childbearing careers, in a population where young women are especially likely to be underweight. These facts are consistent with the interpretations we have presented here — and with our argument that stratification by sibsize can be an important tool in some contexts.

## CRediT authorship contribution statement

**Dean Spears:** Participated in all aspects of this research. **Diane Coffey:** Participated in all aspects of this research. **Jere R. Behrman:** Conceptualization, Writing – review & editing.

## Appendix A Supplementary data

MMC S1 Stata replication files and additional empirical exhibits.

MMC S2

MMC S3

MMC S4

MMC S5

MMC S6

## Footnotes

1 Regressing height-for-age (HAZ, introduced below) on an indicator for having a measured first-born sibling and 119 age-in-months-by sex indicators among Indian third-borns in sibships of 3 yields a coefficient of −0.28 (t>3). Regressing HAZ on an indicator for urban residence and the same age-by-sex controls among the same sample yields a coefficient of 0.30 (t>5). These differences are about twice as large as the overall India-SSA difference.

2 Source: United Nations, Department of Economic and Social Affairs, Population Division. World Population Prospects: The 2017 Revision, custom data acquired via website.

3 Because every variable in Fig. 1 is at the mother or household level, birth order is not a potential confounder of these comparisons.

4 JP report a 1% larger main height sample with 168,108 births. The principal reason that our main height sample does not match their sample exactly is that, to match the birth-order literature, we drop multiple births (such as twins or triplets) throughout our analysis. This follows [7], who explain: “We dropped twins because of the ambiguities involved in defining birth order for twins” (p. 672). In contrast, JP recode these children to share the same birth order; as a result, our final sample is slightly smaller than theirs. Two other differences are that (1) JP cluster standard errors by mother rather than by survey primary sampling units and (2) that in their Eq. (1) JP control for child age only, not child age interacted with sex. When we recreate Table 3 using JP’s treatment of multiple births, age controls, and clustering (not shown), we find results within 1% of a HAZ standard deviation for each coefficient in their main result (with 168,081 observations). We successfully replicate all of our results. We provided this table to referees; it is also available with the Stata files at the journal website as Table A.

5 These are children in the main height sample whose mothers are sterilized or infecund, or whose mothers report wanting no more children. We note that many of the women who report wanting no more children are not using modern contraception. Because high fertility is more common in SSA than in India, over two-thirds of the Indian main height sample is in the “completed fertility” subsample, but less than one-third of the SSA observations are.

6 Even though each stratified household is a balanced panel of two children, adding mother fixed effects yields results that are quantitatively distinct from merely stratifying by sibsize. This is consistent with the possibility that we raise in the next subsection that mother fixed effects reweights the sample (across child ages and birth spacings).

7 JP present further results including triple-interacting birth order, India, and child sex (compare Barcellos et al., 2014); stratifying the sample within India by religion or by state; and interacting covariates with the sex of older siblings. In investigations requested by referees, we find that each of these is fragile to accounting for sibsize. These results are presented as Table C, Table D, and Figure E with the Stata replication files on the journal website. JP also discuss sex-biased fertility stopping (compare Clark, 2000).

8 Column 4 of JP’s main result in Table 2 presents a mother fixed-effects regression with very negative coefficients on birth order × India, implausibly implying that the average gap between first- and third-born siblings is larger than the difference between the children of women who can read whole sentences and women who cannot read at all. In six regressions in Table 3 with mother fixed effects, we find a positive interaction or no evidence of any interaction, including column 8 which simply adds mother fixed effects to our main specification. A *JDE* referee asked us to explain this discrepancy, which we do here. Although we can replicate JP’s mother fixed-effects result when we use their specification, we find that it is fragile. JP parameterize child age in a different way in their mother fixed effects regression than they do in their main specification (Eq. (1) in their text). JP’s fixed-effects result is fragile in three ways: (a) it depends on this particular, alternative age specification but disappears when using the age covariates they use in their Eq. (1) or when using our age-by-sex covariates; (b) preserving the same age covariates in JP’s fixed-effects specification but stratifying the fixed-effects regressions by sibsize also eliminates the negative interaction; (c) preserving the same age covariates in JP’s fixed-effects specification but first residualizing on age in the entire main height sample and then using these residuals in the mother fixed-effects regression (rather than entering age directly as regression controls) changes the result. We provided this table to referees; it is available at the journal website as Table B (see also Spears et al., 2019).
