# Supplementary material for: Endogenous inclusion in the Demographic and Health Survey anthropometric sample: Implications for studying height within households
Source: J Dev Econ. 2022 Mar;155:102783. doi: 10.1016/j.jdeveco.2021.102783 (PMC8857605; doi:10.1016/j.jdeveco.2021.102783)

# Endogenous inclusion in the Demographic and Health Survey anthropometry sample: Implications for studying height within households

## Additional statistical exhibits requested by *JDE* referees

The five exhibits presented here were requested by *JDE* referees. Stata do files posted at the *JDE* website include code for producing these exhibits.

**Table A:** Our Table 3, but now using JP's treatment of multiple births, age control, and clustering (compare JP's Table 2 Column 2, reprinted at left, with our column 1)

**Table B:** JP's mother fixed effects results (compare Column 2 of Panel C here with JP's Column 5 of Table 2) are fragile under any of a number of changes. Dependent variable is HAZ.

**Table C:** Regressions of HAZ on triple interactions including child sex are not robust to any method of accounting for endogenous fertility (Compare with JP's Table 5)

**Table D:** Replication of Table 6, Column 3 from JP shows that it is not robust to accounting for sibsize (dependent variable is HAZ)

**Figure E:** (Compare with JP's Table 4) Within-India split-sample non-parametric means, stratifying by sibsize

**Table A:** Our Table 3, but now using JP's treatment of multiple births, age control, and clustering (compare JP's Table 2 Column 2, reprinted at left, with our column 1)

Dependent variable is HAZ in all columns.

| | (JP's T2C2) | (1) | (2) | (3) | (4) | (5) | (6) | (7) | (8) |
|---|---|---|---|---|---|---|---|---|---|
| **inclusion in sample:** | | | | | | | | | |
| # measured children: | any | any | any | 2 | 2 | 2 | 2 | 2 | ≥ 2 |
| sibsize | any | any | any | any | 2 | 2 | 3 | 3 | any |
| birth orders | any | any | any | any | 1 and 2 | 1 and 2 | 2 and 3 | 2 and 3 | any |
| India | 0.092 | 0.092*** | | 0.0225 | 0.0099 | | -0.263*** | | |
| | (0.018) | (0.018) | | (0.0304) | (0.0310) | | (0.0411) | | |
| birth order 2 × India | -0.144 | -0.149*** | -0.0271 | -0.157*** | -0.0556 | 0.0028 | | | -0.0501 |
| | (0.025) | (0.024) | (0.0312) | (0.0351) | (0.0397) | (0.0395) | | | (0.0357) |
| birth order 3+ × India | -0.377 | -0.374*** | 0.0478 | -0.380*** | | | 0.0532 | 0.135* | -0.0156 |
| | (0.024) | (0.024) | (0.0473) | (0.0390) | | | (0.0542) | (0.0543) | (0.0552) |
| birth order 2 | 0.023 | 0.022 | 0.0184 | -0.079*** | -0.247** | -0.915** | | | -0.20*** |
| | (0.015) | (0.015) | (0.0210) | (0.0232) | (0.0517) | (0.0716) | | | (0.0259) |
| birth order 3+ | -0.066 | -0.065*** | -0.0050 | -0.145*** | | | -0.505** | -1.20*** | -0.407** |
| | (0.013) | (0.013) | (0.0307) | (0.0230) | | | (0.0714) | (0.0998) | (0.0408) |
| sibsize × India | no | no | yes | no | no | no | no | no | no |
| mother fixed effects | no | no | no | no | no | yes | no | yes | yes |
| age-in-months: i.hw1 | yes | yes | yes | yes | yes | yes | yes | yes | yes |
| *n* | 168,108 | 168,081 | 168,081 | 73,362 | 20,097 | 19,842 | 13,692 | 13,528 | 83,235 |

Note: Specifications (1)-(8) match Panel A of our Table 3, other than these three modifications to better match JP's details. Standard errors clustered by mother.

**Table B:** JP's mother fixed effects results (compare Column 2 of Panel C here with JP's Column 5 of Table 2) are fragile under any of a number of changes. Dependent variable is HAZ.

| **inclusion in sample:** | (1) | (2) | (3) | (4) | (5) | (6) | (7) |
|---|---|---|---|---|---|---|---|
| measured children per mother | any | ≥ 2 | ≥ 2 | 2 | 2 | 2 | 2 |
| sibsize | any | any | any | 2 | 2 | 3 | 3 |
| birth orders | any | any | any | 1 and 2 | 1 and 2 | 2 and 3 | 2 and 3 |
| **strategy:** | sibsize control | mother FEs | mother FEs | strat. & FEs | stratification | strat. & FEs | stratification |
| **other notes:** | | | age residualized | | | | |
| **Panel A:** Age is controlled with age-in-months indicators (i.hw1), as in JP's equation (1) and columns 1 and 2 of their Table 2 | | | | | | | |
| India × 2nd born | -0.0105 | -0.0515 | -0.0683 | 0.000981 | -0.0641 | | |
| | (0.0311) | (0.0364) | (0.0365) | (0.0403) | (0.0404) | | |
| India × ≥3rd born | 0.0934 | 0.00142 | -0.0392 | | | 0.123* | 0.0467 |
| | (0.0486) | (0.0576) | (0.0576) | | | (0.0553) | (0.0552) |
| 2nd born | -0.0160 | -0.216*** | -0.125*** | -0.906*** | -0.243*** | | |
| | (0.0214) | (0.0267) | (0.0240) | (0.0733) | (0.0525) | | |
| ≥3rd born | -0.0916** | -0.442*** | -0.255*** | | | -1.168*** | -0.502*** |
| | (0.0319) | (0.0421) | (0.0350) | | | (0.102) | (0.0738) |
| sibsize × India | yes | no | no | no | no | no | no |
| mother fixed effects | no | yes | yes | yes | no | yes | no |
| *n* | 166,153 | 80,785 | 80,785 | 20,374 | 20,374 | 13,864 | 13,864 |
| **Panel B:** Age is controlled with age-in-months indicators × sex (i.hw1##i.b4), our preference, to match the WHO reference tables | | | | | | | |
| India × 2nd born | -0.00659 | -0.0437 | -0.0614 | 0.00962 | -0.0577 | | |
| | (0.0311) | (0.0365) | (0.0365) | (0.0404) | (0.0405) | | |
| India × ≥3rd born | 0.105* | 0.0176 | -0.0251 | | | 0.138* | 0.0610 |
| | (0.0486) | (0.0577) | (0.0577) | | | (0.0557) | (0.0556) |
| 2nd born | -0.0153 | -0.219*** | -0.126*** | -0.919*** | -0.241*** | | |
| | (0.0214) | (0.0267) | (0.0239) | (0.0736) | (0.0527) | | |
| ≥3rd born | -0.0912** | -0.445*** | -0.254*** | | | -1.161*** | -0.496*** |
| | (0.0319) | (0.0421) | (0.0349) | | | (0.102) | (0.0738) |
| sibsize × India | yes | no | no | no | no | no | no |
| mother fixed effects | no | yes | yes | yes | no | yes | no |
| *n* | 166,153 | 80,785 | 80,785 | 20,374 | 20,374 | 13,864 | 13,864 |
| **Panel C:** Age is controlled with age-in-months indicators × India (i.hw1##i.india), as in JP's fixed effects column 5 of their Table 2 | | | | | | | |
| India × 2nd born | 0.0328 | -0.261*** | -0.0751* | -0.0328 | -0.00586 | | |
| | (0.0328) | (0.0481) | (0.0365) | (0.134) | (0.0932) | | |
| India × ≥3rd born | 0.181*** | -0.425*** | -0.0536 | | | -0.201 | 0.0552 |
| | (0.0534) | (0.0861) | (0.0576) | | | (0.196) | (0.139) |
| 2nd born | -0.0281 | -0.180*** | -0.119*** | -0.887*** | -0.275*** | | |
| | (0.0217) | (0.0272) | (0.0240) | (0.0995) | (0.0707) | | |
| ≥3rd born | -0.117*** | -0.369*** | -0.242*** | | | -1.075*** | -0.502*** |
| | (0.0327) | (0.0433) | (0.0350) | | | (0.127) | (0.0920) |
| sibsize × India | yes | no | no | no | no | no | no |
| mother fixed effects | no | yes | yes | yes | no | yes | no |
| *n* | 166,153 | 80,785 | 80,785 | 20,374 | 20,374 | 13,864 | 13,864 |

Standard errors are clustered by survey PSU. All regressions include age controls, as listed in each panel. All regressions implicitly include an India indicator within fixed effects. Column 3 uses, as the dependent variable, residuals from a prior regression (in the entire main height sample) of HAZ on the Panel-specific age specification.

**Table C:** Regressions of HAZ on triple interactions including child sex are not robust to any method of accounting for endogenous fertility (Compare with JP's Table 5)

| | (JP's T5C1) | (1) | (2) | (3) | (4) | (5) | (6) | (7) |
|---|---|---|---|---|---|---|---|---|
| measured children per mother: | any | any | any | ≥ 2 | 2 | 2 | any | any |
| sibsize: | any | any | any | any | 2 | 3 | any | any |
| birth orders: | any | any | any | any | 1 and 2 | 2 and 3 | any | any |
| child sex: | any | any | any | any | any | any | girls only | boys only |
| India | 0.148 | 0.134*** | | | 0.0338 | -0.187** | | |
| | (0.026) | (0.0279) | | | (0.0449) | (0.0594) | | |
| India × girl | -0.111 | -0.0955** | -0.0912* | -0.0553 | -0.0449 | -0.167* | | |
| | (0.036) | (0.0360) | (0.0360) | (0.0726) | (0.0623) | (0.0824) | | |
| India × 2nd born | -0.107 | -0.112** | 0.0239 | -0.0291 | -0.0111 | | -0.0766 | 0.0549 |
| | (0.036) | (0.0353) | (0.0399) | (0.0599) | (0.0609) | | (0.0468) | (0.0462) |
| India × ≥3rd born | -0.352 | -0.357*** | 0.124* | 0.0514 | | -0.0257 | 0.0163 | 0.174* |
| | (0.033) | (0.0341) | (0.0534) | (0.0715) | | (0.0813) | (0.0684) | (0.0718) |
| India × 2nd born × girl | -0.076 | -0.0822 | -0.0680 | -0.0362 | -0.0965 | | | |
| | (0.053) | (0.0512) | (0.0513) | (0.0961) | (0.0953) | | | |
| India × ≥3rd born × girl | -0.051 | -0.0625 | -0.0570 | -0.0798 | | 0.144 | | |
| | (0.047) | (0.0463) | (0.0463) | (0.0856) | | (0.126) | | |
| sibsize × India | no | no | yes | no | no | no | yes | yes |
| mother fixed effects | no | no | no | yes | no | no | no | no |
| child age (i.hw1) | yes | yes | yes | yes | yes | yes | yes | yes |
| *n* | 168,108 | 166,153 | 166,153 | 80,785 | 20,374 | 14,274 | 82,046 | 84,103 |

The leftmost column retypes JP's Column 1 of Table 5 for the reader's comparison. In columns (1) through (7), standard errors are clustered by survey primary sampling unit and 59 age-in-months fixed effects are included.

**Table D:** Replication of Table 6, Column 3 from JP shows that it is not robust to accounting for sibsize (dependent variable is HAZ)

| | (JP's T6C3) | (1) | (2) |
|---|---|---|---|
| India × 2nd born | -0.080 | -0.077 | 0.054 |
| | (0.041) | (0.039) | (0.043) |
| India × ≥3rd born | -0.311 | -0.301 | 0.170 |
| | (0.046) | (0.043) | (0.059) |
| | Many other interaction terms are omitted here for clarity. | | |
| mother fixed effects | no | no | yes |
| | 168, 108 | 166, 153 | 166,153 |

**Figure E:** (Compare with JP's Table 4) Within-India split-sample non-parametric means, stratifying by sibsize

Separating Hindus and Muslims:

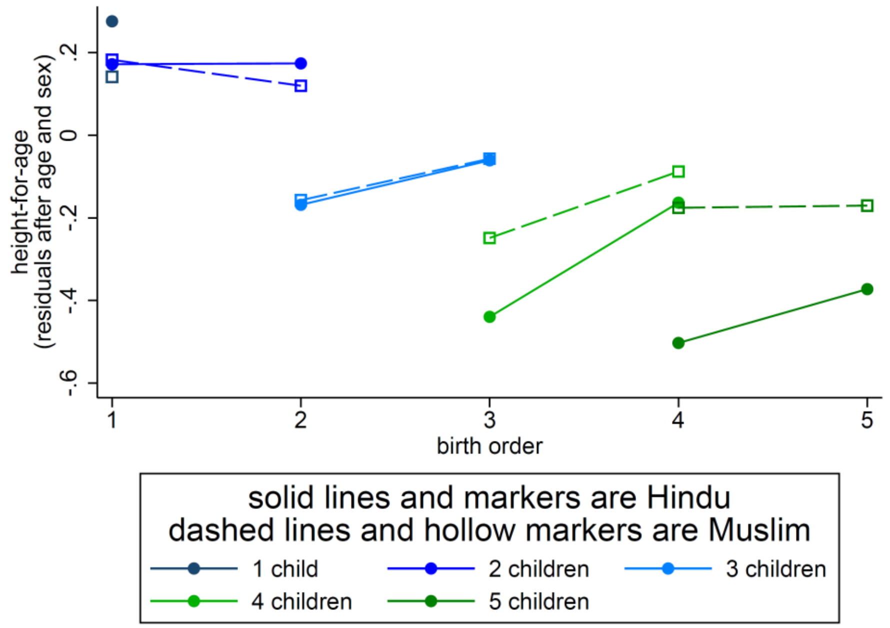


Separating Kerala and the northeastern states from the rest of India:

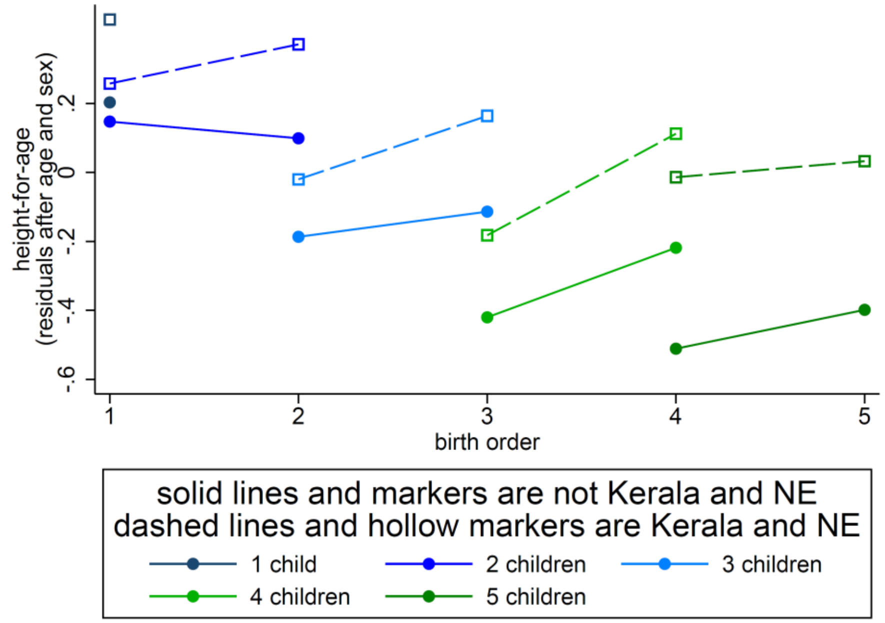

Supplement: MMC S1 — Stata replication files and additional empirical exhibits. [file mmc1.pdf]
