# Supplementary material for: Endogenous inclusion in the Demographic and Health Survey anthropometric sample: Implications for studying height within households
Source: J Dev Econ. 2022 Mar;155:102783. doi: 10.1016/j.jdeveco.2021.102783 (PMC8857605; doi:10.1016/j.jdeveco.2021.102783)
Supplement: MMC S6 [file mmc6.pdf]

Endogenous inclusion in the Demographic and Health Survey anthropometry sample: Implications for studying height within households

README for Stata files

15 October 2021

Exhibit	Location	Do File	Dataset
**Figure 1**	Paper	JDE_RR_main_do_file	DHS_JDE.dta
**Figure 2**	Paper	JDE_RR_main_do_file	DHS_JDE.dta
**Table 1**	Paper	JDE_RR_main_do_file	DHS_JDE.dta
**Table 2**	Paper	JDE_RR_main_do_file	DHS_JDE.dta
**Table 3**	Paper	JDE_RR_main_do_file	DHS_JDE.dta
**Table A**	Online results for JDE referees	JDE_Table_A	DHS_JDE.dta
**Table B**	Online results for JDE referees	JDE_RR_main_do_file	DHS_JDE.dta
**Table C**	Online results for JDE referees	JDE_RR_main_do_file	DHS_JDE.dta
**Table D**	Online results for JDE referees	JDE_RR_main_do_file	DHS_JDE.dta
**Figure E**	Online results for JDE referees	JDE_Figure_E	IABR52FL.dta

DHS Stata files are available for free at measuredhs.com. We use:

- DHS_JDE.dta is our compilation of the relevant variables from 28 DHS survey rounds. Note that line 21 of the do file lists the survey round ("`tab v000`").
- IABR52FL.dta is the simple birth recode file for India only, available from the DHS.

We computed our results using Stata MP 14.2 on the computer detailed on the right (nothing fancy, Dean's five-year-old College of Liberal Arts issue laptop):

THE UNIVERSITY OF TEXAS AT AUSTIN

Latitude E7470

PC name	ECON-D02981
Organization	AUSTIN
Edition	Windows 10 Enterprise
Version	1511
OS Build	10586.1540
Product ID	00329-00000-00003-AA515
Processor	Intel(R) Core(TM) i5-6300U CPU @ 2.40GHz 2.50 GHz
Installed RAM	16.0 GB (15.9 GB usable)

The do file takes about seven minutes. It requires `zscore06`, `reghdfe` and `esttab`.

Change your working directory at the top of the do file. The do file expects that you have subdirectories "data", "logs", and "exhibits" and will look for DHS_JDE.dta in data.

We reprint here the opening lines of the Stata log file which provide details on the data:

```
      name:  DHS_JDE_RR
       log:  C:\Users\spearsde\Dropbox\birth order comment\logs\birth order
DHS for JDE RR.smcl
  log type:  smcl
 opened on:  14 Oct 2021, 05:35:32

.
. version 12.1

. clear all

. set more off

.
. use "data\DHS_JDE"

.
. * These are the DHS survey rounds we use:
. tab v000

    country |
   code and |
      phase |      Freq.     Percent        Cum.
------------+-----------------------------------
        CD5 |     29,548        2.91        2.91
        CG5 |     16,687        1.64        4.55
        CM4 |     29,455        2.90        7.45
        ET4 |     39,881        3.93       11.38
        GH5 |     11,888        1.17       12.55
        GN4 |     27,115        2.67       15.22
        IA5 |    256,782       25.29       40.51
        KE5 |     22,534        2.22       42.73
        LB5 |     22,123        2.18       44.90
        LS4 |     14,708        1.45       46.35
        LS5 |     14,429        1.42       47.77
        MD4 |     20,799        2.05       49.82
        ML5 |     52,140        5.13       54.95
        MW4 |     35,883        3.53       58.49
        NG5 |    104,808       10.32       68.81
        NI5 |     34,378        3.39       72.19
        NM5 |     19,522        1.92       74.12
        RW4 |     30,072        2.96       77.08
        SL5 |     21,136        2.08       79.16
        SN4 |     39,895        3.93       83.09
        ST5 |      7,620        0.75       83.84
        SZ5 |     11,410        1.12       84.96
        TD4 |     21,448        2.11       87.07
        TZ4 |     30,557        3.01       90.08
        TZ5 |     29,777        2.93       93.01
        UG5 |     30,090        2.96       95.98
        ZM5 |     21,366        2.10       98.08
        ZW5 |     19,489        1.92      100.00
------------+-----------------------------------
      Total |  1,015,540      100.00
```

```
.
. * These are the variables we use:
. desc

Contains data from data\DHS_JDE.dta
  obs:     1,015,540
 vars:            20                          6 May 2021 18:03
 size:    32,497,280
-----------------------------------------------------------------------------
              storage   display    value
variable name   type    format     label      variable label
-----------------------------------------------------------------------------
v000            str3    %3s                   country code and phase
v001            long    %12.0g                psu number (national)
v002            int     %8.0g                 household number
v003            byte    %8.0g                 respondent's line number
v011            int     %8.0g                 date of birth (cmc)
v024            byte    %8.0g      LABD       state
v025            byte    %8.0g      LABE       type of place of residence
v119            byte    %8.0g      LABG       household has: electricity
v155            byte    %8.0g      v155       literacy
v312            byte    %8.0g      v312       current contraceptive method
v438            int     %8.0g                 woman's height in centimeters
v445            int     %8.0g      LABAG      body mass index (2 decimals)
v602            byte    %8.0g      v602       fertility preference
bord            byte    %8.0g                 birth order number
b0              byte    %8.0g      b0         child is twin
b3              int     %8.0g                 date of birth (cmc)
b4              byte    %8.0g      LABI       sex of child
hw1             byte    %8.0g                 child's age in months
hw3             int     %8.0g                 child's height in centimeters
hw70            int     %8.0g      LABBU      height/age standard deviation
-----------------------------------------------------------------------------
```
